# Trophic Level Stability-Inducing Effects of Predaceous Early Juvenile Fish in an Estuarine Mesocosm Study

**DOI:** 10.1371/journal.pone.0061019

**Published:** 2013-04-02

**Authors:** Ryan J. Wasserman, Margaux Noyon, Trevor S. Avery, P. William Froneman

**Affiliations:** 1 Department of Zoology and Entomology, Rhodes University, Grahamstown, South Africa; 2 Department of Biology, Acadia University, Wolfville, Nova Scotia, Canada; Aristotle University of Thessaloniki, Greece

## Abstract

**Background:**

Classically, estuarine planktonic research has focussed largely on the physico-chemical drivers of community assemblages leaving a paucity of information on important biological interactions.

**Methodology/Principal Findings:**

Within the context of trophic cascades, various treatments using *in situ* mesocosms were established in a closed estuary to highlight the importance of predation in stabilizing estuarine plankton abundances. Through either the removal (filtration) or addition of certain planktonic groups, five different trophic systems were established. These treatments contained varied numbers of trophic levels and thus different “predators” at the top of the food chain. The abundances of zooplankton (copepod and polychaete), ciliate, micro-flagellate, nano-flagellate and bacteria were investigated in each treatment, over time. The reference treatment containing apex zooplanktivores (early juvenile mullet) and plankton at natural densities mimicked a natural, stable state of an estuary. Proportional variability (PV) and coefficient of variation (CV) of temporal abundances were calculated for each taxon and showed that apex predators in this experimental ecosystem, when compared to the other systems, induced stability. The presence of these predators therefore had consequences for multiple trophic levels, consistent with trophic cascade theory.

**Conclusions/Significance:**

PV and CV proved useful indices for comparing stability. Apex predators exerted a stabilizing pressure through feeding on copepods and polychaetes which cascaded through the ciliates, micro-flagellates, nano-flagellates and bacteria. When compared with treatments without apex predators, the role of predation in structuring planktonic communities in closed estuaries was highlighted.

## Introduction

Trophic interactions play an essential organisational role in community and ecosystem ecology [1]. Biological communities are comprised of numerous species interacting through complex relationships, yet coexisting in equilibrium [2], [3]. Of the many kinds of organisms comprising food webs, top predators are often the most vulnerable to extinction, an aspect of various intrinsic biological traits, including lower population densities and slower reproductive rates [4]. The loss of a top predator could have consequences for a community [5], [6]. As such, predator contributions to community structure have received much attention and their importance has been highlighted in certain biological communities [7], [8], [9], [6], [10], [11]. Predatory top-down control is observed through trophic cascades, across multiple lower trophic levels, underscoring the importance of top predators in food webs and underlying community structure [10], [12].

Empirically, trophic dynamics are generally under-studied and inadequately understood, largely because of the complex nature of community relationships that exist within them [13]. The classic trophic cascade theory originates in the “community” paradigm, whereby linear food chains exist, comprised of distinct trophic levels [8]. Community interactions have however been shown to be much more complex and are more aptly described as food webs with a myriad of trophic relationships that are challenging to identify and disentangle [8], [14], [15]. Characterizing food web interactions are complicated by predation at and on multiple trophic levels [14], [15], [16], competition among species within a trophic level [17], [18], and intra-guild predation, where competing species also engage in predator-prey interactions [14], [19]. In this way, defining planktonic trophic levels is problematic and can perhaps best be described as those organisms within the size limits of a predator's foraging attainability. This attainability varies greatly, however, depending on the planktonic predator [20] and as such, investigating indirect effects of predators across multiple trophic levels is challenging, especially when trying to assess cascade mechanisms. However, detecting cascades is possible without elucidating all mechanisms involved [1].

Within aquatic ecosystems, most demonstrated trophic cascades have been in limnetic environments [5] with comparably few marine and estuarine examples [12]. Furthermore, trophic studies on plankton have either investigated zooplanktivore-zooplankton-phytoplankton interactions [21], [22] or relationships at the microbial level between bacteria and bacterivorous protists [23], [24] with few studies having assessed interactions between these two arenas [25]. When investigating planktonic trophic studies, *in situ* mesocosms are often employed [7], [26], [27].

The use of mesocosms in trophic studies has become increasingly important because they allow investigations of ecological theory without the assumptions and constraints of mathematical and computational methods [28]. Mesocosms are easily manipulated, thus, particularly useful for studying biological interactions across multiple trophic levels and testing specific predator-prey relationships under various environmental conditions [7], [26], [29]. A further advantage is that they can be employed in the field, better simulating natural conditions and mitigating laboratory artefacts [27]. Mesocosms, then, can be broadly defined as stable experimental ecosystem models, generally expected to contain representative subsamples of the system being simulated [30].

Predator-prey relationships are often size-related, with predators being larger than prey [20], [31]. As such, our experimental manipulations involved either the removal of certain size-class planktonic predators using a simple filtration approach, or the addition of macroplanktonic predators, to induce a variety of artificial trophic scenarios. We investigated planktonic trophic level interactions highlighting the presence of trophic cascades in estuarine plankton. We hypothesised that apex-planktonic-predators would stabilize the planktonic community through the maintenance of interactions that transcend multiple trophic levels. Our impetus for this study originates from marine and estuarine food web studies that have mostly focussed on effects of bottom-up services [32], leaving top-down regulatory effects crucial for the understanding of trophic dynamics, largely unknown.

## Materials and Methods

### Ethics statement

All necessary permits for collection and experimentation were acquired for the described field study from the Department of Agriculture, Forestry and Fisheries, Republic of South Africa (permit reference number: RES2011/46). Upon experimental termination, early life-history fish were not released back into the wild, but preserved for further study. Following the “Guidelines for Use of Fishes in Field Research” of the “American Society of Ichthyologists and Herpetologists”, as recommended by Rhodes University, the fish were numbed in ice-water for 1 hour, and then pithed prior to removal of skin samples for use in another study. The fish were then preserved in 10% buffered formalin. Chemical anaesthetic was not used as these chemicals may interfere with natural chemical cues of early life-history predatory fish, essential for the additional study. The procedure used in this study did not require ethical clearance according to the Rhodes University Ethics Committee, who was informed of the study.

### Study site

The study was conducted in the middle reaches of the Kasouga Estuary in the warm-temperate Eastern Cape of South Africa. This medium-sized estuary empties into the Indian Ocean and is located on the south-eastern coastline of South Africa, with a catchment area of 39 km^2^ [33]. Like the vast majority of South African estuaries, the Kasouga Estuary is known as a temporary open/closed system, with the mouth often blocked off from the sea for varied time periods [34], as it was for the duration of the study. At the region near the mouth, when closed, the estuary is roughly 100–200 m in width, narrowing in the upper reaches to between 30–40 m, depending on the season [35]. The approximately 2.5 km of navigable estuary is around 1.8 m deep in the lower and middle reaches and 1.5–2 m in the main channel of the upper reaches [33], [35].

### Experimental set-up

Fifteen 1000 L mesocosm enclosures (1.4 m deep×80 cm wide×80 cm long) constructed of translucent 200 µm thick, virgin polyethylene bags were floated individually in 1.8 m deep water. Each mesocosm was open to the atmosphere at the top, but completely sealed from surrounding waters. The enclosures were secured by a square 80 cm×80 cm frame and covered by a 4 cm×4 cm plastic grid for protection from aerial predators. Frame corners were fitted with airtight 5 L buoys, elevating the top end of the bag from the water's surface by ≈40 cm to ensure no overtopping by estuarine water into the mesocosms during the study. Each mesocosm was secured to a concrete mooring anchored in the estuarine sediment with 50 cm of 10 mm thick elasticated rope to mitigate wave action. Mesocosms were assembled and filled with water after sunset, maximising the incidence of representative taxa including those with diel vertical migrations [36]. All estuarine water was collected on site, into 100 L containers, elevated on the bow of a 3 m long boat, then gravity fed by polyethylene hose into each mesocosm through mesh filters as per trophic treatments.

Five trophic treatments were established (Table 1). Based on a variation of the planktonic predator-prey size ratio theory [20], three treatments (T1–T3) involved the exclusion of size-class plankton, and therefore trophic levels, via filtration of gravity fed water through mesh sizes 20, 80 and 500 µm, respectively. Treatment 4 (T4) used unfiltered water while treatment 5 (T5) contained unfiltered estuarine water with the addition of zooplanktivorous “apex” predators. Early-juvenile freshwater mullet, *Myxus capensis* (Valenciennes, 1836) (31.3±1.72 mm total length) of the Mugilidae family, stocked at natural densities (2 individuals per mesocosm), were employed as the apex predator. Preliminary gut assessments of similar sized individuals from the estuary, showed these fish were indeed practicing zooplanktivory, feeding predominantly on copepods. Fish were captured at the study site using a 25 m seine net with 1 mm mesh on the first evening of the study and were measured and stocked immediately. Triplicate mesocosms were used for each treatment and the entire study was conducted over a 19 day period spanning the new moon.

**Table 1:** Treatment manipulation of mesocosms.

Treatment	Manipulation
T1	Estuary water filtered through 20 µm mesh
T2	Estuary water filtered through 80 µm mesh
T3	Estuary water filtered through 500 µm mesh
T4	Unfiltered estuary water
T5	Unfiltered estuary water, with addition of 2 early life-history fish (natural densities)

Three replicate mesocosms were established for each of the five treatments (T). T1–T3 involved the filtration of gravity fed water through various mesh sized sieves, while T4 was filled with unfiltered estuarine water. For T5, unfiltered estuarine water was employed and stocked with early life-history fish as model apex planktonic predators.

### Physico-chemical and biological sampling

Daily measurements of salinity, temperature (°C) and dissolved oxygen (mg.L^−1^) were recorded from each mesocosm between 14:00 and 15:00 using an Aquaread Aquameter. Biological samples were collected at the start and every third day (day 0, 3, 6, 9, 12, 15 and 18) shortly after physical measurements and during daylight, with the exception of zooplankton samples which were collected after sunset, between 19:30 and 20:30 to capture those organisms that demonstrate diel vertical migrations. Each mesocosm was stirred in a figure of eight pattern using an ore prior to sampling.

Bacterial numbers were estimated by direct counting. Triplicate 1 mL water samples were collected from each mesocosm and preserved with acidified Lugols' iodine following recommendations by Nishino [37]. Samples were gently vacuum filtered at <5 cm Hg through 0.1 µm polycarbonate black membranes, mounted onto glass slides [37], examined under an epifluorescent microscope at ×1000 and bacterial numbers estimated as a mean of triplicate samples [38].

Size fractionated and total chlorophyll-*a* concentrations (Chl-*a*) were determined from a 250 mL water sample collected from each mesocosm. Samples were serially vacuum filtered at <5 cm Hg through 20 µm, 2 µm and 0.7 µm filters and then placed in 8 mL of 90% acetone at −20°C for 24 hours. Chl-*a* concentrations were then determined using fluorometry following the method of Lorenzen [39].

Water (250 mL) for micro- and nano-plankton analysis was collected and preserved using Lugols' iodine solution. At a broad taxonomic level, blue-green algae, dinoflagellates, diatoms, nano-flagellates (<10 µm), micro-flagellates (>20 µm) and ciliates (>20 µm) were identified and enumerated using an Olympus CKX41 inverted microscope at ×400 magnification via the Utermöhl settling technique [40]. Zooplankton was sampled vertically after sunset, using a WP-2 type net with 80 µm mesh size and a 155 mm hoop diameter filtering 26.43 L water for each sampling event. Where possible, zooplankton was identified to the lowest taxonomic level using a Wild M5A stereomicroscope. After day 18, water from the mesocosms of T4 and T5 were filtered through a 1 mm mesh sieve to collect, and determine the abundance of zooplanktonic predators in these treatments.

### Statistical analyses

Temporal variation in population abundances is related to stability [41], [42], [43]; therefore, temporal variability in taxa abundances was employed as the measure of stability, using coefficient of variability (CV) and proportional variability (PV; [44]) as metrics. Definitions of community stability vary [45], [46], [42], [47], [48], but most require some equilibrium point from which differences can be measured [49]. For temporal variability, a proportional metric is typically used with the equilibrium point being the average abundance of the taxon under consideration. Proportional measures provide a degree of independence from the mean unless the underlying dynamics of the system are density dependent, which is typical in ecological systems [50]. Given a mesocosm is essentially a closed population, CV has suitable properties for measuring temporal variability provided there are no (or few) zeros and variability is independent of the mean [51], [50], [44]. However, CV can be biased by zero counts, rare events and other ‘non-normal’ behaviour of population data requiring different indices that allow comparisons across taxa [44]. In contrast to CV, PV is calculated as an average difference in abundance among sampling events and reduces the effects of rare events by comparing all abundances relative to each other rather than to the mean [44].

For each replicate mesocosm, CV was calculated as the standard deviation divided by mean for all days, while PV was calculated as the average proportional difference between all measured abundances at each day [44]. A PV value of 0 equals no change i.e. complete stability, and a value of 1 represents complete instability. More specifically, PV is calculated as: 1) *C* is derived as the number of all possible combinations of sampled abundances in a time series of length *n* (Eq. 1); 2) proportional differences are then calculated as *D*(*z*) (Eq. 2), with *z* expressed as a pair of abundances (*z_i_* and *z_j_*) at any two time steps; and 3) both *C* and *D*(*z*) are then used to calculate PV (Eq. 3) as the average proportional variability of all time steps.
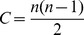
(1)

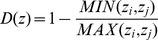
(2)

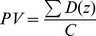
(3)


For each replicate mesocosm, CV and PV values were calculated per taxon and for each treatment as overall CV or PV, i.e. using data from the entire time series (all sampling days). Means (±standard deviations) of the resulting three overall CV and PV values for each treatment were used to compare CV and PV among treatments using ANOVA. Since T5 was assumed to be the most stable or “natural” environment, Dunnett's post-hoc test was used to determine treatment differences with T5 as the reference treatment. PV is a relatively new measure of variability; therefore, CV was presented and compared to provide a reference point for historical studies.

## Results

### Physico-chemical variables

**Figure 1:**
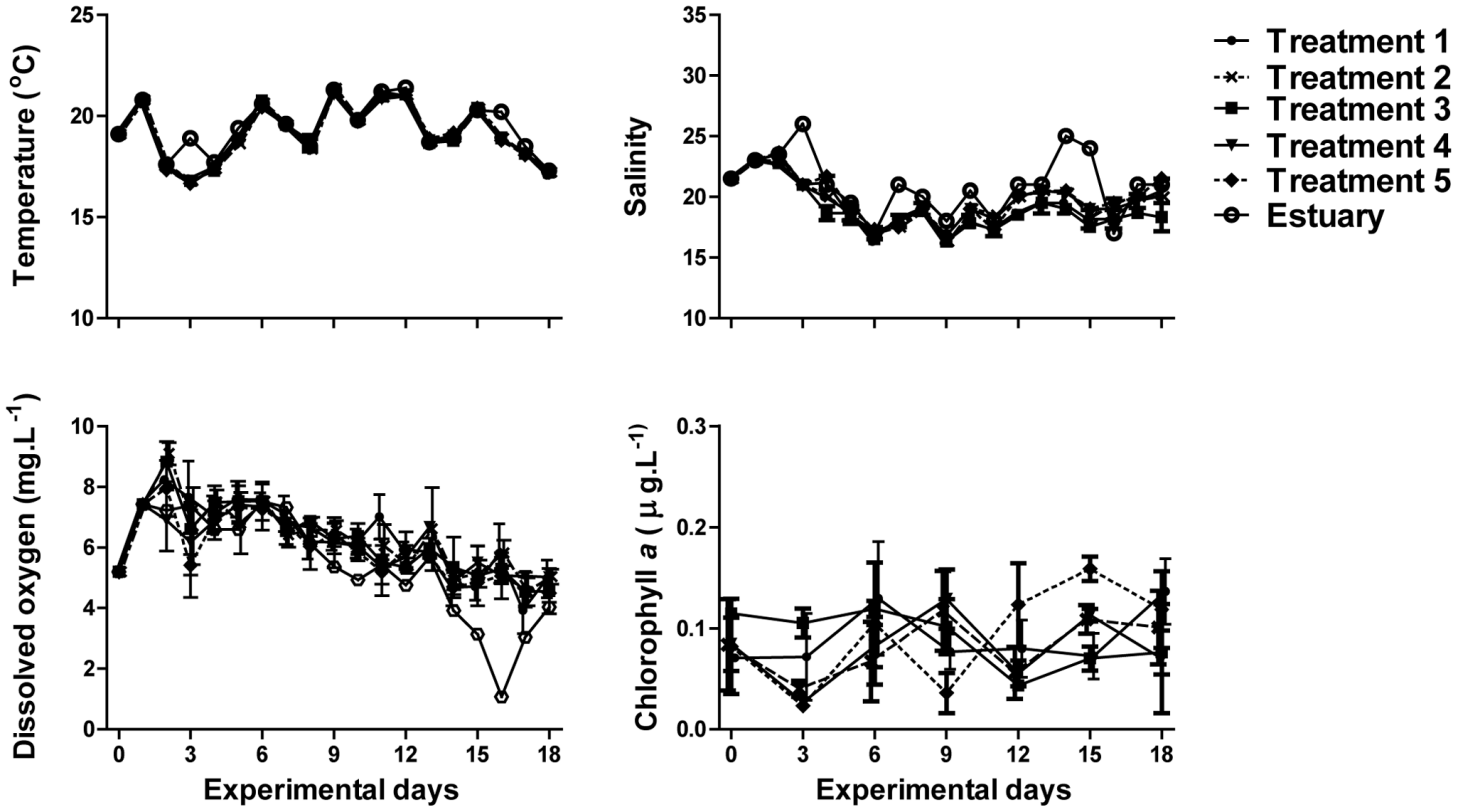
Physico-chemical measurements and chlorophyll-*a* concentrations over time. Mean±standard deviation of temperature, salinity, dissolved oxygen and total chlorophyll-*a* concentrations over time, per experimental treatment and from the estuarine waters at the experimental site. Temperature, salinity and dissolved oxygen measurements recorded daily. Chlorophyll-*a* samples collected every third day.

Temperature and salinity were largely similar across treatments and reflected those values recorded in the estuary (Fig. 1). Initial mesocosm dissolved oxygen levels were however, slightly greater than those within the nearby estuary, with the highest levels being recorded in T1. Nonetheless, the mean (overall) dissolved oxygen values were similar across treatments and remained slightly above those recorded in the estuary (Table 2).

**Table 2:** Physico-chemical parameters of mesocosm and estuary water samples.

	Day 0			All Days (Overall)		
	Temp. (°C)	Salinity	D.O. (mg/L)	Temp. (°C)	Salinity	D.O. (mg/L)
Treatment 1	19.1±0.2	21.7±0.3	7.7±1.2	19.2±1.4	19.1±1.9	6.3±1.2
Treatment 2	19.2±0.1	21.3±0.3	6.7±1.3	19.2±1.4	19.5±1.8	6.3±1.1
Treatment 3	19.0±0.1	21.3±0.3	6.6±0.8	19.2±1.4	18.8±1.9	6.2±1.2
Treatment 4	19.1±0.1	21.7±0.3	6.2±1.1	19.1±1.4	19.7±1.8	6.1±0.9
Treatment 5	19.2±0.1	21.3±0.3	5.4±1.1	19.2±1.4	19.8±1.9	6.0±1.1
Estuary	19.0*	21.5*	5.2*	19.5±1.3	21.1±2.4	5.3±1.7

Initial (Day 0) and overall (All Days) values are presented as mean±standard deviation of 3 replicates. D.O. = dissolved oxygen; *single measurement taken on day of observation.

### Biological samples

The picophytoplankton size fraction (<2.0 µm) dominated the total Chl-*a* concentration within each treatment, followed by the nanophytoplankton (2–20 µm) size fraction (Table 3). Chl-*a* was quite similar among treatments within each size fraction, but decreased in the 2 µm and 20 µm fractions from initial to overall, suggesting that resources in this fraction were being depleted over the study period.

**Table 3:** Fractionated chlorophyll *a* concentrations.

	Day 0 (µg.L^−1^)			All Days (Overall) (µg.L^−1^)		
	0.7 µm	2 µm	20 µm	0.7 µm	2 µm	20 µm
Treatment 1	0.06±0.04	0.08±0.01	0.02±0.01	0.06±0.03	0.03±0.02	0.01±0.01
Treatment 2	0.06±0.01	0.07±0.03	0.02±<0.01	0.06±0.03	0.03±0.02	0.01±<0.01
Treatment 3	0.06±0.01	0.07±0.01	0.02±0.01	0.05±0.03	0.03±0.02	0.01±0.01
Treatment 4	0.06±0.01	0.06±<0.01	0.02±0.01	0.05±0.03	0.03±0.02	0.01±<0.01
Treatment 5	0.07±0.06	0.06±0.01	0.02±<0.01	0.08±0.05	0.03±0.02	0.01±<0.01

Initial (Day 0) and overall (All Days) concentrations recorded as mean±standard deviation of 3 replicates.

Nano-flagellates (<10 µm) numerically dominated the plankton (99.8%), followed by the micro-flagellates (>20 µm) and ciliates (Table 4). In contrast, blue-green algae, diatom and dinoflagellate numerical contributions were minimal. Similarly, of zooplankton sampled with the WP-2 type net, the calanoid copepod, *Pseudodiaptomus hessei* numerically dominated the adult copepod abundance (99.5%) while *Prionospio* sp. dominated polychaete numbers (99.9%) with the vast majority at an early life-history stage. No mortality of stocked young fish occurred during the study as all, and only, the initially stocked young fish were collected at the end of the study. Furthermore, upon filtering mesocosm water at the end of the study, only four isopods (*Exosphaeroma hylecoetes*) were collected from T4 (2 from replicate 1, 1 from replicate 3) and T5 (replicate 2). Therefore, taxonomic groups were broadly defined as: macrozooplanktonic predators (fish), copepods, copepodites and nauplii, polychaetes, ciliates, micro-flagellates, nano-flagellates, and bacteria.

**Table 4:** Mesocosm taxa abundances.

		Treatment 1		Treatment 2		Treatment 3		Treatment 4		Treatment 5	
		Total	Range	Total	Range	Total	Range	Total	Range	Total	Range
Bacteria		4.8^10^	5.7^8^–4.5^9^	4.4^10^	1.0^9^–5.3^9^	4.3^10^	9.1^8^–3.4^9^	4.2^10^	4.8^8^–3.7^9^	3.0^10^	9.8^8^ –1.9^9^
Diatoms	(<10 µm)	135	0–29	160	0–43	168	0–46	145	0–39	35	0–7
Diatoms	(>10 µm)	0	–	0	–	1	0–1	2	0–1	6	0–2
Dinoflagellates	(<10 µm)	30	0–3	32	0–7	30	0–7	30	0–9	14	0–3
Blue-green algae		0	–	1	0–1	2	0–1	1	0–1	2	0–1
Nano-flagellates	(<10 µm)	2.0^5^	4.9^3^–2.1^4^	1.8^5^	4.9^3^–2.2^4^	1.7^5^	2.5^3^–1.8^4^	2.0^5^	3.9^3^–1.7^4^	3.3^5^	1.0^3^–2.2^4^
Micro-flagellates	(>20 µm)	1720	9–204	1264	29–124	1083	4–134	991	9–118	1438	27–120
Ciliates		208	0.7–36.2	194	1–26	132	0–22	208	0–28	190	3–21
Polychaetes											
	*Prionospio* sp.	235	0–78	12085	0–1873	16321	7–1892	13425	3–2285	1390	9–334
	Polychaete spp.	0	–	2	0–1	0	–	0	–	0	–
Nauplii		2376	0–664	1349	0–598	18934	8–3906	11257	4–2040	1757	4–237
Copepodites		539	0–137	286	0–43	5430	0–644	2873	0–312	520	0–49
Copepods											
	*Pseudodiaptomus hessei*	113	0–24	100	0–18	1673	0–238	1308	0–179	97.5	0–13
	*Paracartia longipatella*	0	–	2	0–2	10	0–3	4	0–2	1	0–1
	*Euterpina acutifrons*.	1	0–1	4	0–2	7	0–2	9	0–3	9	0–5
	Cyclopoid spp.	3	0–3	11	0–4	42	0–11	23	0–5	4	0–2
Amphipods											
	Grandidierella sp.	0	–	0	–	0	–	1	0–1	0	–
	Amphipod sp.	0	–	0	–	1	0–1	1	–	0	–
Isopod											
	*Exosphaeroma hylecoetes*	0	–	1	0–1	0	–	1	0–1	2	0–2

Values represent overall abundance and the range (min–max) in values per taxon. Each of bacteria, diatom, dinoflagellate, blue-green algae, flagellate, and ciliate abundances are presented in numbers per mL. All other taxa abundances presented as total numbers per sample (26.43 L).

**Figure 2:**
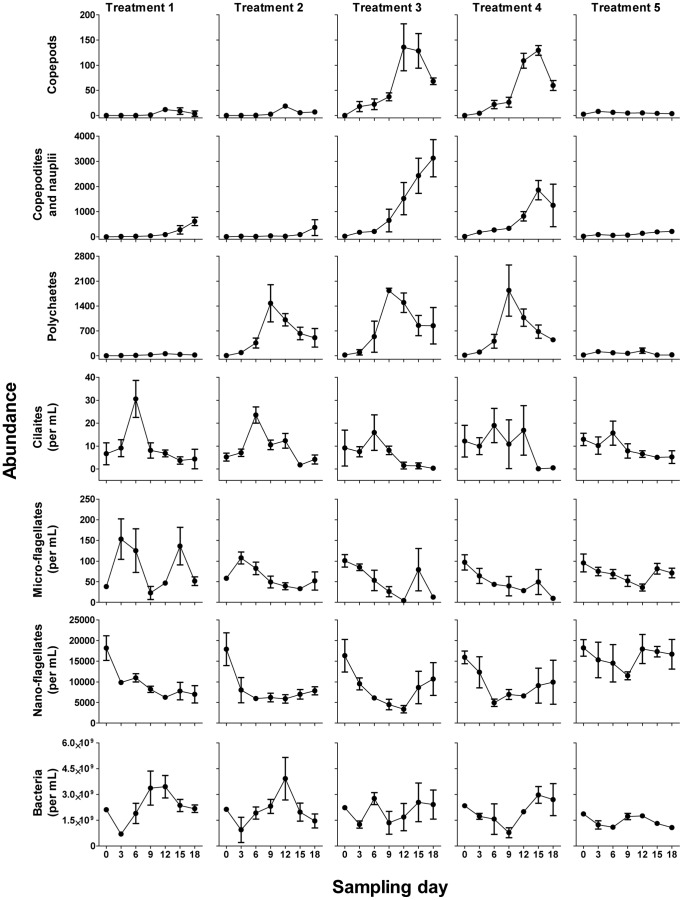
Taxon abundances over time. Bacteria, flagellate and ciliate values are expressed as numbers per mL, whereas polychaete, copepodite and nauplii and copepod values are expressed as numbers per sample (26.43 L). Mean±standard deviation of abundances calculated from three replicates per treatment.

Responses to trophic manipulation were evident in all taxa when comparing abundance patterns among treatments and especially when T1 to T4 are compared with T5 (Fig. 2). The initial lack of copepods in T1 contributed to microplankton (ciliates and micro-flagellates) occupying the top trophic level. This resulted in pronounced and quick increases in abundances of these taxa by day 6, presumably through the released predation pressure by the removal of larger zooplankton. Concomitantly, increased grazing pressure by microzooplankton contributed to a decrease in nano-flagellate abundances and, consequently, an increase in bacterial cell counts.

An increase in polychaete numbers in T2 dampened the initial increase of micro-flagellate and (less so) ciliate abundances, although phytoplankton and bacterial trends were similar to T1, likely because polychaetes were feeding on both the microzooplankton and phytoplankton. The general decrease in micro-flagellate and ciliate numbers in the two last sampling days in T1 and T2 is attributed to the marginal increase of copepod, and copepodite and nauplii abundances. This decrease in micro-flagellates and ciliates probably resulted in a slight increase in nano-flagellate abundance which likely caused a decrease in bacteria abundance.

In general, responses observed in T3 and T4 (*in situ*, but with no fish) were largely similar across taxa. The lack of predation pressure within these two treatments resulted in a steady increase in copepods, copepodites and nauplii numbers over the duration of the study with a noticeable decrease in adult copepods only occurring on day 18. While the abundance of micro-flagellates generally decreased in these two treatments, the ciliates showed an initial increase until about day 6 (T3) and day 5 (T4) before decreasing, potentially highlighting a predator-prey relationship between these two taxa. The polychaete abundance trends in these two treatments were similar to those of copepods, but at an order of magnitude higher, while nano-flagellate abundances showed an inverse trend dropping substantially from day 0 to day 12, then increasing from day 12 to 18. The abundance and variability (standard deviation) of three trophic groups (copepod, copepodites and nauplii and polychaete) in T5 (containing the young zooplanktivorous fish) were low and, relative to other taxa, day-to-day variability was stable over the study period as shown in less variable trends. Again, there was a general decrease in micro-flagellate and ciliate abundances, but these trends were less pronounced than those of other treatments. Finally, nano-flagellate and bacteria abundances in T5 decreased initially, increased over the middle period, and then slowly decreased towards the end of the study. All taxa in T5 had relatively low replicate variability compared with other treatments except for phytoplankton. Nano-flagellate abundance initially decreased until day 9, then increased and stabilized until day 18. Bacterial abundances in T5 were lower and had relatively stable day-to-day variability in comparison with other treatments.

### Statistical analyses

**Figure 3:**
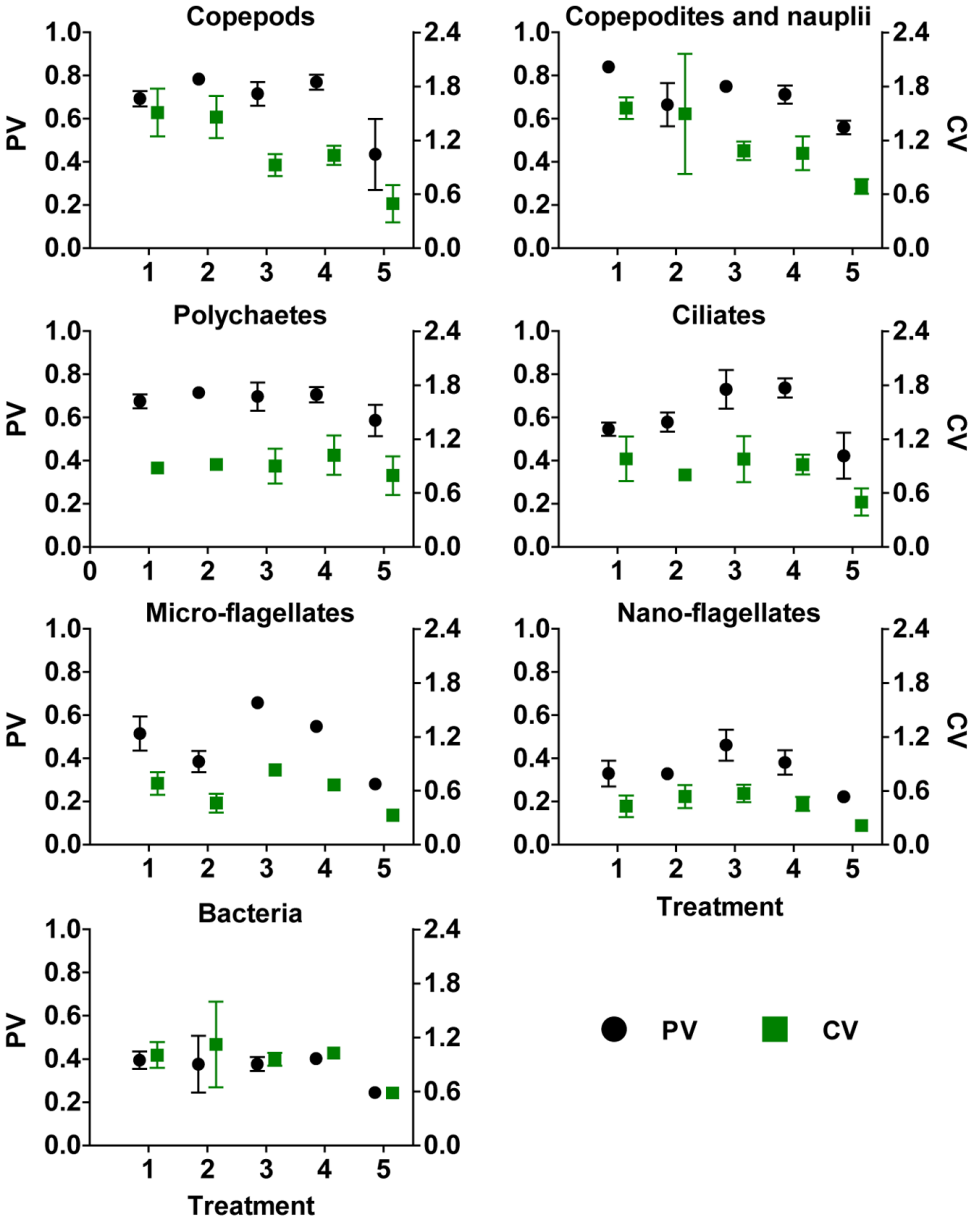
Comparison of the proportional variability (PV) and coefficient of variation (CV) for each taxon. Mean±standard deviation of overall PV and CV calculated from three replicates per treatment. For PV (left y-axis), 0 = complete stability, 1 = complete instability; CV (right y-axis) has no upper bound.

PV values corroborate differences in temporal stability shown in raw abundance values of Figure 2 (Fig. 3). PV values in T5 were consistently the lowest and, thus, more stable over the study period across all treatments. That is, taxa in T5 were less variable overall than taxa in all other treatments and support the observations shown in Figure 2 of less variable abundances. Statistically, 17 of 28 PV comparisons between T5 and the remaining treatments were significantly lower at α = 0.05 and 9 others significantly lower at α = 0.1 (Table 5). CV values confirm PV values and the increased stability (lower PV values) in T5. There was a reduction in significant comparisons with only 13 CV comparisons between T5 and other treatments being significantly lower at α = 0.05 with a further 4 lower at α = 0.1. In general, copepod, ciliates, micro-flagellates, and nano-flagellate differences were upheld between the two metrics, but copepodites and nauplii showed a reversal in significance in treatments T5 vs. T1 and T5 vs. T2, and similar reversals in bacteria were more widespread. Some of the statistical differences found between CV and PV may be attributable to underlying properties of these metrics. However, only a few zeros were present in the entire data set (4%) and zeros did not appear to affect overall abundance trends in those taxa with zero counts in any replicate or on any day. Yet, there was some variation between CV and PV trends. For example, three taxa, copepods, copepodites and nauplii, and ciliates, have CV values that do not follow as closely the pattern of PV values whereas the other taxa show strikingly similar patterns in the two metrics (Fig. 3). The greatest number of zeros was seen in the first 3 sampling days of copepods in T1 and T2, but the CV values in comparison with PV are quite similar for those two treatments suggesting that zeros do not play a role in metric differences. The most important agreement between these metrics is firmly seen in T5 which had the lowest values across all taxa and most of these are significantly lower than other treatments within taxa.

**Table 5:** Dunnett's test results for comparisons between T5 (control) and treatments T1 to T4.

		Copepods	Copepodites and nauplii	Polychaetes	Ciliates	Micro-flagellates	Nano-flagellates	Bacteria
PV								
	5 vs 1	0.002	0.016	0.043	<0.001	<0.001	0.009	0.042
	5 vs 2	0.006	0.004	0.061	0.001	<0.001	<0.001	0.090
	5 vs 3	0.001	0.096	0.029	0.063	0.053	0.079	0.093
	5 vs 4	0.010	<0.001	0.145	0.158	<0.001	0.073	0.052
CV								
	5 vs 1	0.025	0.461	0.316	0.056	0.001	0.037	0.115
	5 vs 2	0.073	0.408	0.845	0.029	<0.001	0.004	0.203
	5 vs 3	<0.001	0.036	0.774	0.191	0.189	0.007	0.050
	5 vs 4	<0.001	0.024	0.917	0.028	<0.001	0.066	0.137

*P* values for proportional variability (PV) and coefficient of variability (CV) are presented for comparisons among treatments. Values in red bold and black bold are α = 0.05 and α = 0.01 respectively.

## Discussion

The stability of copepod abundances in T5 with apex predator and its relative instability in treatments where copepods were not filtered out (T3 and T4), demonstrates the direct effect of stability exerted by the fish on copepods. It is no surprise that the calanoid copepod, *Pseudodiaptomus hessei* dominated the mesozooplankton in our mesocosms, as it often dominates zooplankton abundance and biomass in southern African estuaries [52], [53]. In the absence of fish in T3 and T4, copepod abundance increased which, in turn, negatively impacted micro-flagellate and ciliate abundances, most likely due to increased grazing on these taxa by copepods. This grazing dynamic was consistent with predator-prey cascades [25], [54]. Trophic interactions of ‘larger’ plankton (copepods, micro-flagellates, and ciliates) on smaller phytoplankton was less clear however, and confounded in part by early copepod life stages (copepodites and nauplii) and the presence of polychaetes, which share overlapping prey size distributions with copepods [55], [56], [57]. When copepods increased in abundance, ciliates and micro-flagellate numbers decreased, presumably a result of increased copepod grazing. While the micro-flagellates, ciliates, polychaetes and early life-history stage copepods could be capable of consuming the nano-flagellates [20], [56], [57], the latter would be largely unavailable for direct consumption by the adult calanoids as they are likely smaller than the prey size range of these copepods [55].

Polychaete abundances were also stable in the presence of apex predators and ciliates showed a marked reduction in replicate variability, but were not more stable in T5 over T1. Predator-prey dynamics caused by size fractionation undoubtedly led to trophic pathway shifts possibly preventing ciliates and polychaetes from operating within their predator-prey niche. Caution is, however, required when interpreting these results, as the removal of zooplankton every third day through sampling without reposition of organisms could have affected their overall numbers. While the trends in zooplankton data for most treatments seem to be largely unaffected by the sampling protocol, such removal may have exaggerated the results for selected zooplanktonic components (e.g. metazoans), especially where overall numbers were low. However, the sampling protocol was consistent, and differences in trends across treatments were evident, highlighting the effects of the treatments.

It is evident that removing biological size fractions was effective to limit taxa assemblages within treatments and clear trends in taxa dominance were noted. With the exception of T5 the taxon occupying the top trophic level initially increased in abundance as a result of predator release, and then decreased presumably as a result of density dependent factors or resource depletion. In some cases two taxa showed this pattern, e.g. copepods and polychaetes in T3, or ciliates and micro-flagellates in T1 (although not as pronounced for micro-flagellates), suggesting these respective taxa were not engaged in predator-prey interactions, at least over this period. In T1, ciliates and micro-flagellates initially occupied the top trophic level and increased dramatically over the first few days. In turn, they decreased nano-flagellate and bacterial abundances. When *Prionospio* polychaetes were included, as in T2, they appeared to dominate predation on micro-flagellates and potentially grazed on phytoplankton [57]. Very little change was seen in ciliate abundances with polychaetes present, suggesting that polychaetes did not feed on ciliates. Ciliates likely grazed on nano-flagellates and when ciliate abundances decreased in a consistent way (as in T3 and T5), nano-flagellate abundances increased. Where ciliates became variable, nano-flagellate abundances were also variable (see T4), suggesting that there was a strong coupling between ciliates and nano-flagellates. While intra-guild predation has been shown to exist between ciliates and micro-flagellates [58], competitive interactions may also be present because both have the ability to feed on nano-flagellates and bacteria [20]. However, despite indiscriminate grazers having effects on numerous taxa and some predator-prey relationships being more complex than simple one-to-one observations, we have successfully demonstrated that successively higher trophic levels affect lower trophic levels in a cascading fashion [8], [9]. Furthermore, proportional variability was shown to be a powerful analysis for teasing out the stabilizing role of predators on prey abundances, highlighting some of these cascading effects.

Ecological investigations have long emphasized the importance of physical processes, so called “bottom up effects”, in structuring aquatic ecosystems [59]. In marine research the focus is often on the implications of various physico-chemical characteristics in structuring planktonic food webs [32]. Since the present study focused entirely on biological interactions, the study required physico-chemical homogeneity across treatments. Indeed, salinity and temperature measurements were consistently similar across treatments and comparable to that of the estuary. Furthermore, despite the initial differences in dissolved oxygen concentrations across treatments, likely resulting from increased aeration during the filtration procedure, the overall concentrations were similar. Particle aggregation and sinking, resulting in an export of materials from mesocosm water columns were, however, not measured during the study. While such information would be useful, potential differences in material export among treatments would likely be a result of the biological manipulations, rather than physico-chemical differences. As such, through physico-chemical homogeneity the significance of biological interactions in structuring ecosystems could be characterized, and the importance of predator-prey interactions highlighted.

A fundamental element of any manipulative trophic-interaction analysis is the adequate observation of predator numbers for the duration of the study [60]. Since there is often a relative equilibrium of coexistence between predators and their prey in natural systems [2], [3], it is necessary to establish a stable apex predatory pressure in experimental scenarios such as in the present study, where natural states were simulated (T5). The reproductive cycle of our apex predators precluded rapid reproduction; therefore, only the initial stocked young fish were present throughout the study and were healthy. As such, verified stable apex predation pressure was qualified for T5, allowing for comparison with treatments whereby the top of the food web was less stable over time, ultimately highlighting the presence of trophic cascades.

In the oligotrophic warm- temperate Kasouga Estuary, the biological interactions between the taxa were not exclusively predator-prey in nature and as such, the exposition of cascade mechanisms *per se* proved elusive. However, the presence of trophic cascading was evident across treatments, where multiple lower trophic levels were affected, regardless of the varied dominant taxa at the top trophic level. The presence of the apex predator in this system provided a consistent pressure, stabilizing copepod and polychaete numbers, and furthermore, through various cascade mechanisms, also stabilized ciliate, micro-flagellate, nano-flagellate and bacterial abundances. As such, we have shown that young fish can assume the role of apex planktonic predators, mediating interactions and stability at multiple lower trophic levels in oligohaline estuary environments.
